# Multivariate analyses of risk factors associated with laboratory exposure incidents

**DOI:** 10.14745/ccdr.v48i78a06

**Published:** 2022-07-07

**Authors:** Maryem El Jaouhari, Nicole Atchessi, Rojiemiahd Edjoc, Megan Striha, Samuel Bonti-Ankomah

**Affiliations:** 1 Health Security Regional Operations Branch, Public Health Agency of Canada, Ottawa, ON

**Keywords:** laboratory exposures, laboratory-acquired infections, risk factor, human pathogens and toxins

## Abstract

**Background:**

Laboratories involved in the study of pathogenic biological agents pose an inherent risk of exposure to the laboratory workforce and the community. Laboratory biosafety and biosecurity activities are fundamental in minimizing the likelihood of unintentional exposure incidents. The objective of this study is to describe the factors that are associated with the occurrence of exposure incidents in a laboratory setting through a predictive model.

**Methods:**

The Laboratory Incident Notification Canada is a nationally mandated surveillance system that gathers real-time data from submitted reports of laboratory incidents involving human pathogens and toxins. Data on laboratory exposure incidents were extracted from the system between 2016 and 2020. The occurrence of exposure incidents per month was modelled using a Poisson regression with several potential risk factors, including seasonality, sector, occurrence type, root causes, role and education of people exposed and years of laboratory experience. A stepwise selection method was used to develop a parsimonious model with consideration of the significant risk factors identified in the literature.

**Results:**

After controlling for other variables in the model, it was found that 1) for each human interaction related root cause, the monthly number of exposure incidents was expected to be 1.11 times higher compared to the number of incidents without human interaction (*p*=0.0017) as a root cause and 2) for each standard operating procedure-related root cause, the monthly number of exposure incidents was expected to be 1.13 times higher compared to the number of incidents without a standard operating procedure related root cause (*p*=0.0010).

**Conclusion:**

Laboratory biosafety and biosecurity activities should target these risk factors to reduce the occurrence of exposure incidents. Qualitative studies are needed to provide better reasoning for the association of these risk factors with the occurrence of exposure incidents.

## Introduction

Laboratory work involving the study of biological agents poses an inherent risk of exposure to the laboratory personnel and the community. Although laboratory biosafety and biosecurity guidelines have advanced considerably, there is still a need to guide risk mitigation decisions to target the most important risks that are associated with exposure incidents ((1)).

There were several risk factors identified in the literature that are associated with exposure incidents in a laboratory, with the most significant factors being human errors ((2,3)). Evidence from case report studies has shown that the common risk factors associated with exposure incidents occurrence are improper use of personal protective equipment ((4–6)), insufficiently trained staff ((7,8)) and fewer years of work experience ((9)). Other case report studies found that high-risk work tasks ((9)) and working with needles ((4,10,11)) were also associated with the occurrence of exposure incidents. In addition, risk factors identified in a case-control study and a cross-sectional study included a lack of standard operating procedures (SOP) ((12)) and inadequate biosafety risk assessments ((13)), respectively.

Although these studies are important for identifying trends in the occurrence of exposure incidents, case report studies may not be generalizable to all laboratory settings. Additionally, many of these studies use descriptive statistics to identify the risk factors which have the potential to produce biases ((2,3,6,9,13)). These studies were mostly cross-sectional or case reports and may not capture the most important risk factors contributing to exposure incidents. To adequately mitigate risks, it is critical to prioritize risk factors involved in exposure incidents that have been identified through surveillance over a longer period of time. To identify the significant risk factors that are associated with an increase or decrease in exposure incidents, inferential analyses using existing surveillance data over a long period of time are warranted.

In this report, surveillance data were analyzed and a mathematical model that predicts the risk factors that are associated with exposure incidents was developed. This model could inform licensed facilities to prioritize laboratory biosafety and biosecurity activities on important risk factors to reduce the occurrence of exposure incidents in the future.

## Methods

### Data sources

The Laboratory Incident Notification Canada (LINC) surveillance system collects real-time data from licensed laboratory incidents involving human pathogens and toxins. It is the only mandated surveillance system that is required to collect exposure incidents from licensed laboratories across Canada. Notification and follow-up reports of laboratory incidents are received through the Biosecurity Portal and then captured by the internal Customer Relationship Management system.

Exposure incidents were defined as those with the potential to cause infection/intoxication or had resulted in a suspected or confirmed laboratory-acquired infection involving human pathogens and toxins that are within the scope of the *Human Pathogens and Toxins Act* ((14)) and the *Human Pathogens and Toxins Regulations* ((15)).

Data were extracted from this system on the exposure incidents that took place from January 1, 2016, to December 31, 2020. Incidents that did not have a known occurrence date were also included if they were reported during this period. Data of the most recent follow-up reports were used for analysis, while the data of initial reports were used where corresponding follow-up reports and/or data were not present as of the data extraction date, February 8, 2021. The extracted data were cleaned and inspected for any missing values, duplicate entries and/or outliers.

### Data analysis

The data from the LINC surveillance system was imported into SAS EG 7.1 to perform data manipulation and multivariate analyses. The original database contained 284 rows collected over five years, where each row contained one incident. One incident can involve multiple occurrence types and more than one root cause can be identified for one incident. Data were transformed to obtain a monthly count of exposure incidents and to examine their seasonality occurrence over five years. In this transformation, 284 individual exposure incidents were grouped by month to give 60 monthly observations. The sample size was smaller due to the transformation from reported incidents per row to reported incidents per month per row. Three months were excluded from our sample because there were no incidents reported. The final dataset had 57 observations.

A Poisson regression was used to model the occurrence of exposure incidents per month because count data is not normally distributed. Using a stepwise selection method, the following independent variables were analyzed: seasonality (year, month); monthly count of sector (hospital, academic, government, environmental, private, public health, veterinary); occurrence type (animal related, equipment, insect, loss containment, personal protective equipment, procedure, sharp, spill, unknown, other); root causes (training, communication, equipment, human interaction, management, SOP, other); role (technician, student, researcher, manager, animal handler, other); education of person exposed (high school, technical, university degree); and route of exposure (inhalation, inoculation, absorption, other). The monthly number of affected persons as well as their median years of laboratory experience using the monthly data points were also included in the analysis.

Both univariate and bivariate analyses were first conducted to explore the associations between the predictive independent variables and the outcome variable of interest. Independent significant parameters identified in the bivariate analyses were included in the multivariate Poisson regression analysis. A *p*-value of 0.05 was chosen as the cut-off point for entry and exit into the stepwise procedure. Stepwise selection of variables was conducted by groups of variables to identify factors associated with the occurrence of exposure incidents because of the high number of variables and small sample size.

## Results

From 2016 to 2020, there were 614 individuals exposed in the 284 confirmed exposure incidents reported to LINC. The average monthly occurrence of incidents was 4.98. Laboratory characteristics of the exposure incidents can be found in **Table 1**. In this dataset, the median years of laboratory experience was 7.25. Most exposed individuals had a technical/trade diploma (66.3%) or a bachelor’s degree (25.5%) and belonged in the hospital sector (57.5%), academic (17.7%) or private (11.2%) sectors. Most individuals exposed were technicians/technologists (74.9%). Among exposed individuals, the most common route of exposure to human pathogens and toxins was through inhalation (62.2%) or inoculation (14.2%). The most commonly reported occurrence types were procedural (23%) and sharps-related (22.0%). Standard operating procedures (25.6%) and human interactions (19.4%) were the most commonly cited root causes. Additional descriptive data on exposure incidents may be found in our annual reports between 2016 and 2020 ((2,16–19)).

**Table 1:** Descriptive and bivariate analyses of all predictive variables of exposure incidents

Variables	Exposure incidents		Coefficient	*p*-value
	n	%		
Root cause (N=679)				
Training	72	10.6	1.28	<0.0001
Communication	73	10.8	1.35	<0.0001
Equipment	84	12.4	1.32	<0.0001
Human interaction	132	19.4	1.22	<0.0001
Management	75	11.0	1.37	<0.0001
SOP	174	25.6	1.22	<0.0001
Other	69	10.2	1.24	0.0001
Occurrence type (N=378)^a^				
Animal related	17	4.5	1.29	0.0077
Equipment	23	6.1	1.25	0.0002
Loss containment	18	4.8	1.55	<0.0001
PPE	45	11.9	1.27	<0.0001
Sharp	83	22.0	1.30	<0.0001
Procedure	87	23.0	1.28	<0.0001
Spill	45	11.9	1.40	<0.0001
Unknown	11	2.9	1.11	0.4005
Other	49	12.9	1.27	<0.0001
Role (N=614)				
Technician	460	74.9	1.03	<0.0001
Student	58	9.4	1.25	0.0001
Researcher	18	2.9	1.21	0.0185
Animal handler	7	1.1	1.25	0.1714
Manager	15	2.4	1.22	0.0058
Other	56	9.1	1.15	<0.0001
Sector (N=273)				
Hospital	95	34.8	1.27	<0.0001
Academic	101	37.0	1.26	<0.0001
Environmental	2	0.7	1.43	0.1955
Private	29	10.6	1.15	0.0671
Public health	29	10.6	1.38	<0.0001
Veterinary	10	3.7	1.10	0.5149
Other government	7	2.6	1.42	0.0292
Education (N=510)				
High school	42	8.2	1.09	<0.0001
Technical	338	66.3	1.03	<0.0001
University (Bachelor's degree)	130	25.5	1.05	0.0001
Route of exposure (N=614)				
Inoculation	87	14.2	1.32	<0.0001
Inhalation	382	62.2	1.02	<0.0001
Absorption	48	7.8	1.29	<0.0001
Other	97	15.8	1.02	0.0226
Years of experience (Median)	7.25	N/A	1.01	0.6020

Abbreviations: N/A, not applicable; PPE, personal protective equipment; SOP, standard operating procedure

^a^ No exposure incidents were insect-related

Bivariate regression analysis results can also be found in Table 1. The relationship between the outcome of interest (number of exposure incidents per month) and each independent variable was determined through Poisson regression. The exponents of the estimated regression coefficients and the *p*-values are listed in Table 1.

Multivariate Poisson regression analyses for the association between the number of exposure incidents and predictive independent variables are shown in **Table 2**. The exponents of the estimated regression coefficients and the *p*-values are listed in Table 2. With consideration of the significant risk factors identified in the literature, a parsimonious model was developed, which included the following predictive variables: human interaction and SOP issues as root causes; and roles (including students and technicians). The analyses revealed that having a role as a student or as a technician/technologist in the laboratory was not significantly associated with the number of exposure incidents per month. It was found that for each human interaction and SOP related root cause, the monthly number of exposure incidents is expected to be 1.11 times higher (*p*=0.0017) compared with the occurrence of incidents without human interaction as a root cause, after controlling for other variables in the model. It was also found that for each SOP related root cause, the monthly number of exposure incidents is expected to be 1.13 times higher (*p*=0.0010) compared with the occurrence of incidents without an SOP related root cause, after controlling for other variables.

**Table 2:** Multivariate analysis of exposure incidents by risk factors using Poisson regression (Model 1)

Parameter	Coefficient^a^	SE	Coefficient (95% CI)	*p*-value
Student	1.04	0.0584	0.92, 1.16	0.5488
Technician	1.00	0.0055	0.99, 1.01	0.6444
Human interaction	1.11	0.0347	1.04, 1.19	0.0017
SOP	1.13	0.0362	1.05, 1.21	0.0010

Abbreviations: CI, confidence interval; SE, standard error; SOP, standard operating procedure

^a^ The exponents of the estimated regression coefficients after controlling for other variables

Bivariate Poisson regression analyses for the association between the number of exposure incidents and seasonality in **Table 3**. The exponents of the estimated regression coefficients and the *p*-values are listed in Table 3. The analyses revealed that the month of June was significantly associated with less occurrence of exposure incidents when compared with December (*p*=0.0286).

**Table 3:** Bivariate analysis of exposure incidents by seasonality using Poisson regression (Model 2)

Parameter (month)^a^	Exponent (estimate)	SE	Exponent (95% CI)	*p*-value
January	0.89	0.2928	0.50, 1.58	0.6987
February	0.98	0.2849	0.56, 1.72	0.9496
March	0.86	0.2782	0.50, 1.48	0.5795
April	0.75	0.2887	0.43, 1.32	0.3190
May	1	0.2673	0.59, 1.69	1.000
June	0.45	0.3684	0.22, 0.91	0.0286
July	1	0.2673	0.59, 1.69	1.000
August	0.82	0.2814	0.47, 1.43	0.4845
September	1.07	0.2628	0.64, 1.79	0.7929
October	0.86	0.2782	0.51, 1.48	0.5795
November	0.93	0.2724	0.55, 1.58	0.7855

Abbreviations: CI, confidence interval; SE, standard error

^a^ Reference category=December

## Discussion

Our primary objective for this study was to identify the risk factors that were associated with exposure incidents occurrence in laboratory settings through a predictive model. Multivariate Poisson regression analyses revealed that human interaction and SOP related root causes were significantly associated with the occurrence of exposure incidents. The monthly number of exposure incidents was also found to be significantly lower in June through bivariate analyses.

Through descriptive analysis, previous studies identified 1) lack of awareness of or compliance with SOP and 2) human interactions as leading root causes ((2,3,12)); however, our study provided adjusted estimates that quantify and confirm the contribution of these causes to the increase in exposure incidents. Human interaction was commonly described as, but not limited to, a violation (cutting a corner, not following correct procedure, deviating from SOP) or an error (a mistake, lapse of concentration, or slip of some sort) ((18)). Standard operating procedure-related issues were described as documents not being followed correctly for the task, or as SOP not being in place ((18)).

Technicians and technologists were commonly identified as those more often involved in exposure incidents when compared with other individuals in the laboratories ((2,3,20)). These previous findings were based on descriptive statistics and might be explained by the high number of technicians and technologists working in laboratory settings ((2,3,20)); however, the multivariate model of our study highlighted that the contribution of the role of technicians to the increase in exposure incidents was not significant, when the other variables remain constant. Risk mitigation decisions in licensed facilities should mainly target human interaction and lack of compliance to SOP, to prevent the occurrence of exposure incidents.

Contrary to the widespread evidence that work experience is correlated with risk of errors ((21)), our study did not find an association between the median years of experience and the increase in exposure incidents. This result could be due to the lack of granularity of the work experience variable that summarizes the years of experience of all affected people during a given month.

When considering seasonality as a factor involved in the occurrence of exposure incidents, our results revealed that the month of June had significantly lower occurrence of exposure incidents. It is unclear why there are fewer exposure incidents during this month; however, a potential explanation could be a smaller laboratory workforce during the summer due to summer vacations, and thus a reduced number of human interactions and consequently a reduced number of exposure incidents.

The results from this study could be used to inform licensed facilities about the factors that are associated with exposure incidents so that adequate measures are implemented to minimize the likelihood of exposure incidents. Human interactions, non-compliance with SOP and seasonality are important factors to consider for reducing the occurrence of exposure incidents; however qualitative research is required to better understand these findings. A qualitative study would provide insights into why these factors contribute to exposure incidents and how they can be properly addressed in laboratory settings to avoid or reduce exposure incidents.

### Strengths and limitations

The main strength of this study is the use of inferential statistics and multivariate models to identify the risk factors associated with the occurrence of exposure incidents. The majority of previous studies use descriptive statistics to identify the risk factors that have the potential to introduce biases because of potential confounding variables. The use of descriptive statistics is also limited since they do not take into account the relationships between variables, and can therefore only be used to describe and report observations. With the use of inferential analyses, we were able to determine which factors contributed significantly to the occurrence of exposure incidents and also the magnitude of their effects through a predictive model. This study also benefited from the use of existing national surveillance data over a longer period compared with previously published articles, allowing for more accurate identification of the most significant risk factors that predict the occurrence of exposure incidents. Our predictive model could inform licensed facilities to prioritize laboratory biosafety and biosecurity activities on the risk factors identified to reduce the occurrence of exposure incidents in the future.

The most significant limitation of this study was the low sample size due to the transformation of the data into monthly data, which was required to conduct the multivariate analyses and to examine the seasonality. In addition, the LINC surveillance system only captures the information on the affected people and not for the entire laboratory workforce. The information on the entire laboratory workforce could be valuable to compare the characteristics of those who are exposed and those who are not. Furthermore, the surveillance system does not collect sufficient data on all potential predictive variables. For example, the system collects data on management oversight; however, additional information on the role of management oversight in controlling biosafety and biosecurity risks in the laboratories could be valuable.

## Conclusion

This study found that human interactions and SOP-related issues were significantly associated with the occurrence of exposure incidents. These findings are also consistent with the literature, which emphasizes the need for licensed facilities to examine current safety protocols regarding compliance to SOP and human interactions. Additional research, such as qualitative studies, is needed to provide better reasoning for the association of these risk factors with the occurrence of exposure incidents.
